# Prebiotic Potential of Oligosaccharides and Polysaccharides Extracted from *Leucaena leucocephala* Seeds

**DOI:** 10.3390/foods15111890

**Published:** 2026-05-27

**Authors:** Viviane da Silva Sousa Almeida, Amanda Graziela Gonçalves Mendes, Carmem Duarte Lima Campos, Laís Araújo Souza Wolff, Ariadina Jansen Campos Fontes, José Lima Pereira-Filho, Taynara Figueiredo Costa, Cinara Regina Aragão Vieira Monteiro, Alan Silva de Menezes, Harvey Alexander Villa Vélez, Kátia Danielle Araújo Lourenço Viana, Valério Monteiro-Neto

**Affiliations:** 1 Laboratory of Basic and Applied Microbiology, Center of Biological and Health Sciences, Federal University of Maranhão, Sao Luis 65080-805, MA, Brazil; viviane.almeida@discente.ufma.br (V.d.S.S.A.); amanda.graziela@discente.ufma.br (A.G.G.M.); carmem.campos@discente.ufma.br (C.D.L.C.); jlp.filho@discente.ufma.br (J.L.P.-F.); taynara.fc@discente.ufma.br (T.F.C.); 2 Nucleus of Basic and Applied Immunology, Center for Biological and Health Sciences, Federal University of Maranhão, Sao Luis 65080-805, MA, Brazil; lais.wolff@discente.ufma.br; 3 Laboratory of Immunophysiology, Center of Biological and Health Sciences, Federal University of Maranhão, Sao Luis 65080-805, MA, Brazil; ariadina.jansen@discente.ufma.br; 4 School of Physical Therapy, Florence University Center, Sao Luis 65020-490, MA, Brazil; cinara.monteiro@florence.edu.br; 5 Department of Physics, Center of Exact and Technological Sciences, Federal University of Maranhão, Sao Luis 65080-805, MA, Brazil; alan.menezes@ufma.br; 6 Graduate Program in Environmental Science and Technology, Center of Exact and Technological Sciences, Federal University of Maranhão, Sao Luis 65080-805, MA, Brazil; harvey.villa@ufma.br; 7 School of Nutrition, Center of Biological and Health Sciences, Federal University of Maranhão, Sao Luis 65080-805, MA, Brazil; katia.viana@ufma.br

**Keywords:** *Leucaena leucocephala*, soluble fibers, oligosaccharides, polysaccharides, functional food

## Abstract

**Background/Objectives:** Plant-derived soluble fibers are being explored as sustainable prebiotic ingredients; however, tropical legumes such as *Leucaena leucocephala* remain understudied. This study evaluated soluble fibers from *L. leucocephala* seeds after simulated gastrointestinal digestion, focusing on rheological properties, microbial selectivity, metabolite production, and intestinal safety. **Methods:** The anatomical parts of the seed underwent INFOGEST 2.0 digestion. Soluble fibers were characterized by GC-MS monosaccharide profiling, viscosity, and SEM/EDS analyses, and were used as substrates for both probiotic and pathogenic bacteria. Fermentation supernatants were analyzed for short-chain fatty acids and lactate, and cytotoxicity was assessed using Caco-2 cells. **Results:** Endosperm polysaccharides exhibited high apparent viscosity (>300 cP) and pseudoplastic behavior. Monosaccharide profiles revealed the presence of galacto-oligosaccharides and arabinoxylo-oligosaccharides in the oligosaccharide fraction, and galactomannans, xylans, and arabinoxylans in the polysaccharide fraction. Polysaccharides selectively promoted the growth of *Lacticaseibacillus rhamnosus* GG and *Bifidobacterium* spp., comparable to or exceeding that of fructo-oligosaccharides (*p* < 0.05), while limiting pathogenic bacteria. Fermentation produced acetate and lactate concentrations of >4500 ppm and >1000 ppm, respectively. Caco-2 viability remained >90% across all treatments. **Conclusions:** Compartment-resolved analysis identified the endosperm as the principal source of digestion-resistant viscous fiber, selectively fermented by probiotic bacteria at levels matching or exceeding fructo-oligosaccharides. These findings position *L. leucocephala* endosperm fiber as a candidate prebiotic substrate, warranting further preclinical evaluation.

## 1. Introduction

According to the International Scientific Association for Probiotics and Prebiotics (ISAPP), a prebiotic is a substrate selectively utilized by host microorganisms that confers a health benefit [1]. In this context, dietary fibers such as oligosaccharides and polysaccharides are among the main candidates for prebiotic activity because they resist enzymatic digestion in the upper gastrointestinal tract and reach the colon, where they are fermented by bacteria of the gut microbiota, including *Bifidobacterium* spp. and *Lactobacillus* spp., leading to the production of short-chain fatty acids (SCFAs) such as acetate, propionate, and butyrate [1,2]. These metabolites support host health by modulating immune responses and promoting intestinal and systemic homeostasis [3].

SCFAs perform complementary functions. Acetate serves as an energy substrate and regulates lipid metabolism [4], whereas propionate participates in hepatic gluconeogenesis and glycemic control [5]. Butyrate, in turn, serves as the primary energy source for colonocytes, strengthens the intestinal barrier, and exerts anti-inflammatory effects [6,7]. Moreover, SCFAs inhibit histone deacetylases and promote regulatory T-cell differentiation, thereby supporting immune tolerance [8].

*Leucaena leucocephala*, native to Central America and Mexico, stands out among tropical legumes for its high productivity and its adaptation to tropical and semi-arid environments [9,10]. Its seeds contain proteins, unsaturated lipids, fibers, and bioactive carbohydrates, including galactomannans and α-galactosides [11,12]. Although *L. leucocephala* seeds are primarily used in animal nutrition, their use in human consumption among rural populations has also been reported. In this context, the seeds are thermally processed, such as by boiling or roasting, and then milled into flour [11]. Beyond this general composition, *L. leucocephala* seeds exhibit diverse anatomical and functional compartmentalization, with the endosperm enriched in galactomannan-type reserve polysaccharides and the cotyledons enriched in proteins and lipids. This organization is relevant because seed galactomannans are associated with thickening, water-binding, and viscosity-building properties in food systems, as well as increased digesta viscosity, delayed gastric emptying, and slower glucose diffusion during digestion [11,13,14,15].

In this context, seeds from tropical plants have been increasingly studied as sources of functional compounds with potential prebiotic effects. Tropical seeds such as dates and coix, as well as legumes such as soybeans and sesame, exhibit selective fermentability, stimulating the growth of beneficial bacterial species and increasing SCFA production in vitro [16,17,18].

*L. leucocephala* seeds have been characterized compositionally and for the techno-functional properties of their galactomannans, but the prebiotic potential of their soluble fiber, after gastrointestinal digestion, has not been examined. Previous studies have also not resolved how the seed coat, cotyledon, and endosperm contribute differently to the fiber pool, leaving open the question of which seed compartment should be targeted for human applications. This study addresses both gaps. We applied INFOGEST 2.0 digestion separately to each anatomical part and combined rheological characterization, SEM/EDS surface and elemental analysis, GC-MS monosaccharide profiling, fermentation by probiotic and pathogenic bacteria, short-chain fatty acid and lactate quantification, and Caco-2 cytocompatibility within a single experimental framework. Pathogen assays were performed in a synthetic culture medium developed for this purpose (Brazilian patent application BR 10 2025 025261-9), which removes the carbon-source background of conventional rich broths and allows substrate selectivity to be tested directly.

## 2. Materials and Methods

### 2.1. In Vitro Simulated Digestion of Seed Anatomical Parts

Each anatomical part of the seeds (1 g) was subjected to thermal pre-treatment in distilled water at 100 °C for 20 min to facilitate the separation of the seed’s anatomical structures. This was followed by soaking in distilled water for 24 h at 4 °C to ensure complete imbibition of the seed tissues, facilitate manual separation of the seed coat, cotyledons, and endosperm, and limit microbial growth during the prolonged soaking step. After separation, the samples were then subjected to in vitro simulated digestion using the INFOGEST 2.0 protocol [19] at 37 °C to simulate the physiological conditions of the human gastrointestinal tract across three sequential phases: oral, gastric, and intestinal.

For the oral phase, the samples were mixed with simulated salivary fluid (SSF) containing KCl (15.1 mM), KH_2_PO_4_ (3.7 mM), NaHCO_3_ (13.6 mM), MgCl_2_·6H_2_O (0.15 mM), and uric acid (0.34 mM), with the pH adjusted to 7.0 using HCl or NaOH. α-Amylase (200 U/mL, Sigma-Aldrich, St. Louis, MO, USA) and CaCl_2_ (0.75 mM) were added, and the final volume was adjusted to 20 mL with SSF. The mixture was incubated at 37 °C for 2 min under constant agitation.

For the gastric phase, simulated gastric fluid (SGF) was added, containing KCl (6.9 mM), KH_2_PO_4_ (0.9 mM), NaHCO_3_ (25 mM), NaCl (47.2 mM), MgCl_2_·6H_2_O (0.1 mM), CaCl_2_ (0.075 mM), and pepsin (2000 U/mL, Sigma-Aldrich). The pH was adjusted to 3.0 with HCl or NaOH. The final volume was 40 mL. Digestion was carried out for 2 h at 37 °C under constant agitation.

For the intestinal phase, simulated intestinal fluid (SIF) was prepared using the same salts as SGF, except for NaHCO_3_ (85 mM), and with the addition of bile salts (10 mM). Pancreatin (providing 100 U/mL of trypsin activity, Sigma-Aldrich) and CaCl_2_ (0.6 mM) were added, and the pH was adjusted to 7.0 with HCl or NaOH. The final volume was adjusted to 60 mL with SIF. The mixture was incubated for 2 h at 37 °C under constant agitation. At the end of the digestive process, the viscosity of the resulting fluids was immediately measured.

### 2.2. Determination of Viscosity

The apparent viscosity of the fluids obtained after in vitro digestion was determined for four samples: digestive fluids from the cotyledons, digestive fluids from the seed coat, digestive fluids from the endosperm, and the fluid from the commercial prebiotic fructooligosaccharide (FOS), used as a reference prebiotic compound. The analysis was performed by rotational viscometry using a Brookfield DV-II+ Pro viscometer (Brookfield Engineering Laboratories, Middleboro, MA, USA) equipped with spindle LV3-63 (Spindle No. 63), operating in rotational mode with temperature control at 25 °C.

The sample volume used for each measurement was 300 mL, sufficient to fully submerge the spindle in a Griffin-type beaker. Viscosity was measured using the fluid collected at the end of the simulated digestion process, after completion of the oral, gastric, and intestinal phases, ensuring that the analysis reflected the rheological properties of the final digestive matrix. Measurements were taken at progressive rotational speeds ranging from 10 to 200 rpm (10, 20, 30, 40, 50, 60, 70, 80, 90, 100, 120, 150, and 200 rpm). Apparent viscosity values (centipoise, cP) were automatically recorded by the viscometer (model DV-II+ Pro, Brookfield, USA), along with their respective error percentages.

Apparent viscosity data were modeled using the Power Law (μ = *k*·γ*^n^*^−1^), as described by Wang and Cui [20], to determine the consistency index (*k*) and the flow behavior index (*n*), thereby characterizing the rheological properties of the digestive fluids. All analyses were performed in triplicate.

### 2.3. Extraction of Soluble Fibers

The extraction procedure was performed using the digestive fluid collected after the simulated digestion process, which included the oral, gastric, and intestinal phases. Following viscosity analysis, samples were centrifuged at 10,000× *g* for 15 min, and the insoluble material was discarded. Ethanol (92.8%) was added at a 1:4 (*v*/*v*) ratio to precipitate polysaccharides [21,22]. The precipitated material was separated by filtration through nylon cloth with an estimated pore size of 150 µm [22,23] to separate macromolecules such as polysaccharides [24]. The resulting supernatant was centrifuged again at 10,000× *g* for 15 min to recover the oligosaccharide fiber.

The control extraction, performed without simulated digestion, followed Viana’s protocol [25] with adaptations. Anatomical seed parts were homogenized in distilled water (1:26, *w*/*v*) for 10 min at room temperature using a high-speed rotary blender until a viscous solution formed. Samples were centrifuged at 10,000× *g* for 15 min, and the insoluble material was discarded. Ethanol (92.8%) was added at a 1:4 (*v*/*v*) ratio to precipitate polysaccharides. Filtration was performed using nylon cloth with a 150 µm pore size to isolate polysaccharides. The supernatant was then centrifuged at 10,000× *g* for 15 min to obtain oligosaccharides. Both control and digested samples were dried in a forced-air oven (Marconi MA035, Piracicaba, Brazil) at 80 °C for 3 h until a constant weight was reached.

### 2.4. Scanning Electron Microscopy (SEM) and Energy-Dispersive X-Ray Spectroscopy (EDS)

Morphological and elemental analyses were performed on soluble fibers obtained before simulated digestion and on soluble fibers extracted and dehydrated after simulated digestion. Morphological characterization was conducted using a scanning electron microscope (SEM) (EVO 15, Zeiss, Oberkochen, Germany). Images were collected at accelerating voltages of 9.0–15.0 kV using a secondary electron detector in high-vacuum mode. Dried samples were mounted on aluminum stubs using conductive carbon tape and sputter-coated with gold.

The elemental composition of the sample surfaces was determined by energy-dispersive X-ray spectroscopy (EDS) coupled to the SEM system, using a Bruker X-Flash 410 detector (Bruker, Billerica, MA, USA). Elements were identified from the spectra and reported as mass percentages.

### 2.5. Monosaccharide Analysis of Fibers After Simulated Digestion

The monosaccharide composition of soluble fibers obtained after simulated digestion was determined by gas chromatography coupled with mass spectrometry (GC-MS), using an Agilent 7890B gas chromatograph coupled to a 5977A mass selective detector (Agilent Technologies, Santa Clara, CA, USA). Both polysaccharide and oligosaccharide samples (~1 mg) were hydrolyzed with 0.3 mL of 2 M trifluoroacetic acid (TFA) at 120 °C for 2 h. After cooling to room temperature, residual acid was removed by sequential methanol washes, followed by evaporation under a nitrogen stream in an ice bath. Additional isopropanol washes were performed to ensure complete removal of TFA.

The released monosaccharides were derivatized with 0.1 mL of N-trimethylsilyl imidazole (TMSI) at 70 °C for 1 h to enhance their volatility and stability for detection. The derivatized samples were diluted with 150 µL of methanol and injected into the GC-MS system. Separation was performed on a Supelco Equity-1 capillary column (Supelco, Bellefonte, PA, USA) using a temperature program that included an initial step at 80 °C for 2 min, followed by a 2 °C/min increase to 180 °C with a 2 min hold, and a final ramp of 30 °C/min to 250 °C with a 5 min hold.

Mass spectra were compared with commercial standards of glucose, fructose, galactose, mannose, arabinose, and xylose, which had been injected previously to establish retention times and confirm identification, as described by Black et al. [26] and Zhu et al. [27]. These data were used to determine the monosaccharide composition of the soluble fibers obtained after digestion and to infer the oligosaccharides and polysaccharides in the material.

### 2.6. Cytotoxicity Assay Using Human Intestinal Cells (Caco-2)

The oligosaccharide and polysaccharide fibers extracted after in vitro digestion were subjected to cytotoxicity assays to evaluate their safety in human intestinal epithelial cells. The analysis was performed using the Caco-2 cell line, recognized as a representative model of the intestinal barrier in toxicological and food bioactivity studies [28]. As a functional reference, FOS, which have been validated as a safe prebiotic without cytotoxic effects on intestinal epithelial cells, were used [29].

Cell viability was assessed using the MTT assay (3-(4,5-dimethylthiazol-2-yl)-2,5-diphenyltetrazolium), as described by Mosmann [30] with adaptations. Cultures of Caco-2 cells in supplemented Dulbecco’s Modified Eagle Medium [DMEM—supplemented with 20% fetal bovine serum (FBS), penicillin G (60 mg/L), and streptomycin (100 mg/L)], were maintained at 37 °C in a humidified atmosphere with 5% CO_2_ until confluence. After trypsinization, cells were counted using a Neubauer chamber and seeded into 96-well plates at 10^5^ cells/well (200 μL).

After 24 h of adhesion, cells were treated with fiber suspensions at concentrations of 50, 100, 200, 400, and 800 µg/mL for 24, 48, and 72 h. FOS was tested at the same concentrations. The negative control consisted of cells cultured in medium without treatment. Following incubation, the medium was removed, and 200 µL of fresh medium containing 10 µL of MTT solution (5 mg/mL in PBS) was added. Plates were incubated for 3 h at 37 °C, protected from light. The supernatant was then discarded, and 100 µL of dimethyl sulfoxide (DMSO) was added to solubilize the formazan crystals. Absorbance was measured at 580 nm using a microplate reader (Spectra Max 190, Molecular Devices, San Jose, CA, USA). Cell viability was expressed as a percentage relative to the negative control.

### 2.7. In Vitro Fermentation Assay with Beneficial Microorganisms

The bacterial species used in the fermentation assays were *Lacticaseibacillus rhamnosus* GG (ATCC 53103), *Limosilactobacillus fermentum* (ATCC 23271), and *Bifidobacterium animalis* subsp. *lactis* (HN019), and *Bifidobacterium breve* (ATCC 15700). Cultures were grown in de Man, Rogosa, and Sharpe (MRS) broth at 37 °C for 24 h under anaerobic conditions.

Fermentation media were prepared by adding the soluble fibers (oligosaccharides and polysaccharides) and the following controls: glucose, used as a rapidly assimilable carbon source; FOS, used as a reference prebiotic; a medium without carbohydrate (negative control); and a medium without carbohydrate but inoculated with the bacterial species to confirm the absence of growth in the absence of a carbon source. All substrates were autoclaved at 121 °C for 15 min and tested at concentrations of 0.5, 1.0, 1.5, and 2.0% (*w*/*v*).

Cultures were inoculated to a final concentration of 1 × 10^6^ CFU/mL and incubated at 37 °C under anaerobic conditions. Bacterial growth was monitored over time by measuring optical density at 600 nm at 0, 24, and 48 h [31]. All experiments were performed in triplicate.

### 2.8. In Vitro Fermentation Assay with Pathogenic Microorganisms

Four pathogenic or potentially pathogenic bacterial species were selected to assess the selectivity of *L. leucocephala* fibers, covering distinct taxonomic groups, oxygen requirements, and pathological relevance: *Escherichia coli* (ATCC 25922) (aerobic/facultative), a representative member of Enterobacteriaceae whose overgrowth is associated with intestinal dysbiosis; *Staphylococcus aureus* (ATCC 25923) (aerobic/facultative), a Gram-positive pathogen that may proliferate under compromised gut barrier function; *Enterococcus faecalis* (ATCC 29212) (aerobic/facultative), an opportunistic pathogen commonly involved in nosocomial infections that can emerge under immunosuppressive conditions; and *Clostridium butyricum* (ATCC 860) (obligate anaerobe), a species of particular interest due to its dual status as both a probiotic (e.g., strain MIYAIRI 588) and a potential pathogen, depending on strain-specific toxin production and host context. This combination enables a rigorous assessment of microbial selectivity according to the ISAPP criteria for prebiotic classification [1], as it challenges fibers with pathogens that occupy different ecological niches and possess distinct carbohydrate-utilization machinery, ranging from facultative Proteobacteria to strict anaerobic Firmicutes. The facultative bacterial species were cultured in Mueller-Hinton (MH) broth at 37 °C for 24 h, and *C. butyricum* was cultured in sodium thioglycolate broth at 37 °C for 24 h.

For the fermentation assays, a chemically defined medium was prepared containing sodium chloride, ammonium sulfate, a vitamin mix, mineral salts, and essential amino acids, according to the formulation described by Pereira et al. [32]. For *C. butyricum*, sodium thioglycolate was included, and after cooling, 0.5 M L-cysteine was added. The pH of all media was adjusted to 7.2 using 0.1 mol/L NaOH. For the fermentation assays, soluble fibers, oligosaccharides, and polysaccharides were tested separately, with each substrate added to the medium at 2% (*w*/*v*) (equivalent to 2 g per 100 mL) as the sole carbon source. The assays were compared with positive controls (glucose and FOS and a negative control (medium without carbohydrate) to confirm the absence of growth in the absence of a carbon source.

Cultures were inoculated at a final concentration of 1 × 10^6^ CFU/mL and incubated at 37 °C for 24 h under aerobic conditions for *E. coli*, *S. aureus*, and *E. faecalis*, and for 48 h under anaerobic conditions for *C. butyricum*. Bacterial growth was monitored by measuring optical density at 625 nm. All experiments were conducted in triplicate.

### 2.9. Short-Chain Fatty Acid Analysis of Bacterial Fermentation Supernatants

The fermentation supernatants were collected after 24 h of incubation and filtered through a 0.22 µm membrane filter. Short-chain fatty acids (acetic, propionic, and butyric acids) and lactic acid were quantified by ion chromatography with conductivity detection using a 930 Compact IC Flex system (Metrohm AG, Herisau, Switzerland) equipped with a Metrosep Organic Acids 250/7.8 column (Metrohm AG, Herisau, Switzerland). The analysis was performed in a column furnace at 35 °C, using 0.5 mmol/L sulfuric acid as the mobile phase at a flow rate of 0.500 mL/min and 20 mmol/L lithium chloride as the chemical suppression solution. Identification and quantification were based on retention times and external calibration curves constructed from analytical standards. The standards used were lactic acid (Neon, 84.6% purity), acetic acid (Neon, 99.9% purity), propionic acid (Neon, 99.4% purity) (Neon, Sao Paulo, Brazil), and butyric acid (Sigma-Aldrich, 99% purity). Detection was performed by conductivity.

### 2.10. Statistical Analysis

Statistical analyses were performed using SigmaPlot version 15 (Systat Software Inc., Chicago, IL, USA). Results were presented as mean ± standard deviation or as median with interquartile range (P25–P75), depending on data distribution. Rheological parameters were analyzed using the Kruskal–Wallis test, followed by Dunn’s post hoc test for multiple comparisons. Cell viability (MTT assay) data were analyzed using one-way analysis of variance (ANOVA) followed by Tukey’s post hoc test, or the Kruskal–Wallis test followed by Dunn’s post hoc test. Growth of probiotic bacterial species was evaluated using three-way repeated-measures ANOVA, with substrate, concentration, and incubation time as factors, followed by Tukey’s post hoc test. Growth of pathogenic bacterial species was analyzed using one-way ANOVA followed by Tukey’s post hoc test, or the Kruskal–Wallis test followed by Dunn’s post hoc test when appropriate. Comparisons of elemental composition and fiber yield before and after simulated digestion were performed using Student’s *t*-test. For all analyses, differences were considered statistically significant at *p* < 0.05.

## 3. Results

### 3.1. Rheological Properties of Digested Fluids

**Figure 1:**
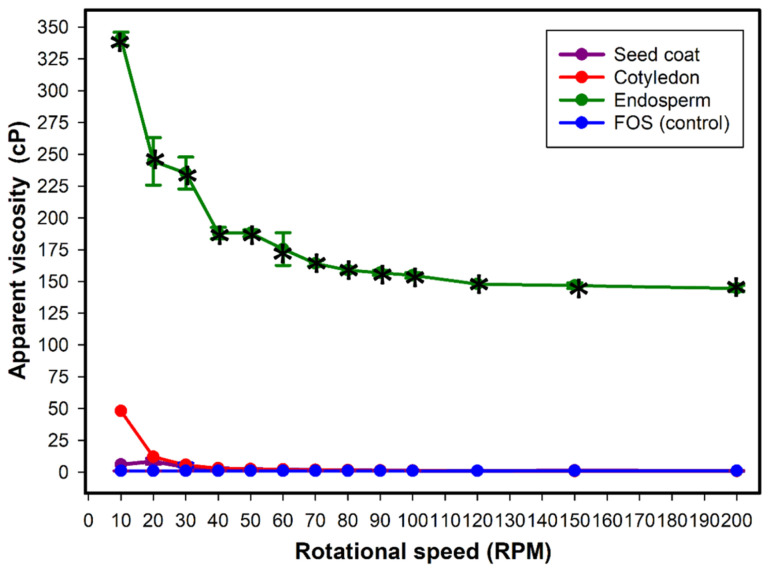
Apparent viscosity (cP) of digested fluids from the endosperm, cotyledon, and seed coat of *L. leucocephala* seeds, compared with the prebiotic control FOS, as a function of rotational speed (rpm). Data are presented as means ± standard error (*n* = 3). The endosperm fluid shows pronounced pseudoplastic behavior, characterized by a progressive reduction in viscosity with increasing shear rate, whereas the cotyledon, seed coat, and FOS display low, relatively constant viscosity values across the evaluated rotational range. Asterisks (*) indicate differences relative to FOS (*p* < 0.05).

Among the digested fluids, only the endosperm showed higher viscosity than the prebiotic control FOS (*p* < 0.05). The endosperm fluid exhibited apparent viscosities above 300 cP at lower rotational speeds (10 and 20 rpm), whereas the digested fluids from the seed coat and cotyledons remained below 50 cP, comparable to the control (*p* > 0.05). Figure 1 illustrates the progressive reduction in viscosity of the endosperm fluid as rotational speed increased, indicating pseudoplastic flow behavior, whereas the viscosities of the seed coat and cotyledon fluids remained relatively constant across the evaluated rotational range, characteristic of Newtonian behavior.

Among the digested samples, the endosperm had the highest consistency index (*k* = 574.662), indicating greater resistance to flow at low shear rates (Table 1). In contrast, the seed coat and cotyledon showed substantially lower *k* values and shear-thinning behavior, consistent with their low and nearly constant apparent viscosity across the evaluated shear range.

### 3.2. Yield of Soluble Fibers After Simulated Digestion

The simulated digestion of the anatomical parts of *L. leucocephala* seeds revealed distinct yields of soluble fiber among the analyzed samples (Table 2). The endosperm showed the highest yield, with 62.0% polysaccharides before digestion and 56.0% after digestion (*p* = 0.025), indicating a reduction in fiber mass after digestion. The seed coat exhibited a moderate yield, with 10.8% soluble fiber before digestion and 9.0% after digestion (*p* = 0.311). The cotyledon showed the lowest soluble fiber content, with 0.2% both before and after digestion (*p* = 0.326).

Overall, the endosperm was the primary source of soluble polysaccharides in *L. leucocephala* seeds, followed by the seed coat, whereas the cotyledon contributed minimally to the total soluble fiber content.

### 3.3. Morphological Changes in Fibers After Digestion According to Scanning Electron Microscopy (SEM)

**Figure 2:**
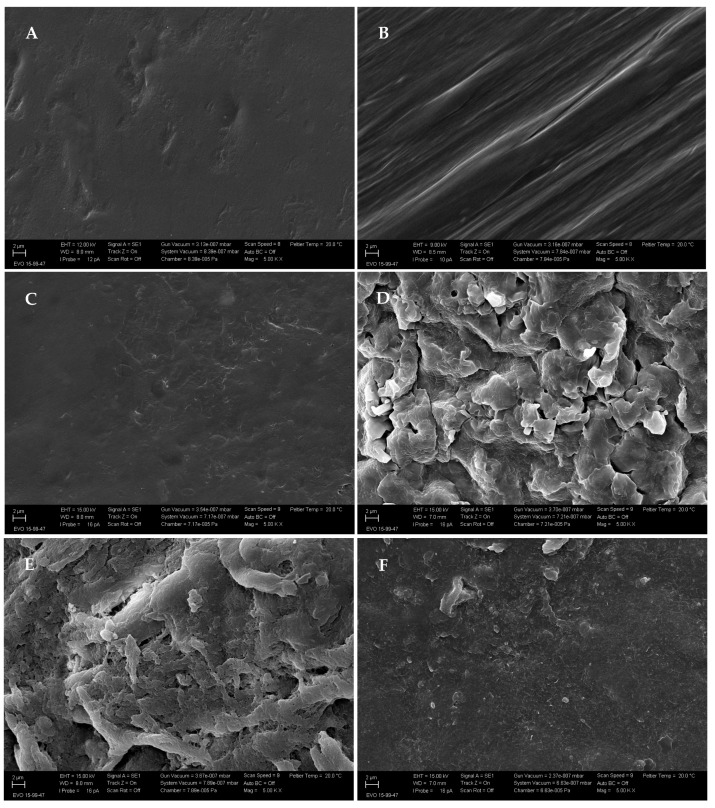
Morphological changes in soluble fibers isolated from *L. leucocephala* seeds before and after in vitro simulated digestion. (**A**) Seed coat before digestion. (**B**) Seed coat after digestion. (**C**) Cotyledon before digestion. (**D**) Cotyledon after digestion. (**E**) Endosperm before digestion. (**F**) Endosperm after digestion. Images were obtained by scanning electron microscopy (SEM). Scale bars = 2 µm, magnification = 5000×.

SEM analysis revealed morphological changes in soluble fibers before and after simulated digestion (Figure 2). These changes included surface disorganization, fissures, and alterations in matrix texture, with distinct patterns observed among the seed’s anatomical parts.

The oligosaccharide fibers obtained from the seed coat before digestion (Figure 2A) exhibited a compact and continuous surface without visible fissures. After digestion (Figure 2B), fibrillar structures with partial exposure of internal layers were observed, indicating modification of the external matrix.

The oligosaccharides obtained from the cotyledons before digestion (Figure 2C) showed a smooth and homogeneous surface. After digestion (Figure 2D), the matrix exhibited discontinuities, cavities and increased porosity, reflecting structural alteration following enzymatic exposure.

The polysaccharides extracted from the endosperm before digestion (Figure 2E) displayed an irregular surface with agglomerates and heterogeneous texture. After digestion (Figure 2F), the material showed a more uniform and continuous surface, with reduced irregularities and no evident fissures or matrix disruption.

### 3.4. Elemental Composition of Soluble Seed Fiber Tissues Before and After In Vitro Digestion

Elemental analysis by SEM-EDS revealed changes in the surface composition of the seed coat, cotyledon, and endosperm of *L. leucocephala* seeds after simulated gastrointestinal digestion (Table 3). Digestion was associated with lower levels of elements associated with the organic matrix, particularly carbon and nitrogen, with variations observed among the seed’s anatomical parts.

In the seed coat, carbon content decreased (*p* < 0.001), whereas calcium increased (*p* < 0.001). Potassium (*p* = 0.016) and magnesium (*p* = 0.016) also increased, whereas oxygen decreased (*p* = 0.012) compared with the undigested sample. The cotyledon showed pronounced compositional changes, with reductions in carbon (*p* < 0.001) and nitrogen (*p* < 0.001), accompanied by lower levels of calcium (*p* = 0.002), oxygen (*p* < 0.001), and magnesium (*p* = 0.089). In the endosperm, digestion resulted in lower carbon content (*p* < 0.001) and higher levels of calcium (*p* = 0.001) and potassium (*p* < 0.001), whereas oxygen (*p* = 0.109) and nitrogen (*p* = 0.543) showed comparable values before and after digestion.

### 3.5. Monosaccharide Characterization and Structural Inference

GC-MS analysis following acid hydrolysis and trimethylsilyl (TMS) derivatization revealed distinct monosaccharide profiles in oligosaccharides and polysaccharides isolated from *L. leucocephala* seeds (Table 4).

In the oligosaccharide hydrolysates, galactose, arabinose, and xylose were the predominant monosaccharides detected. This monosaccharide composition is consistent with the presence of galactooligosaccharides (GOS) associated with galactan-rich regions and arabinoxylooligosaccharides (AXOS) related to arabinoxylan structures.

In the polysaccharides, mannose, galactose, xylose, and arabinose were detected. The simultaneous occurrence of mannose and galactose indicates galactomannan-type polysaccharides. In addition, the detection of xylose alone or in combination with arabinose indicates the presence of xylan-type domains with xylopyranose backbones and arabinoxylan regions containing arabinose substitutions.

### 3.6. Oligosaccharide and Polysaccharide Fibers Showed Low Cytotoxicity to Intestinal Caco-2 Cells

**Figure 3:**
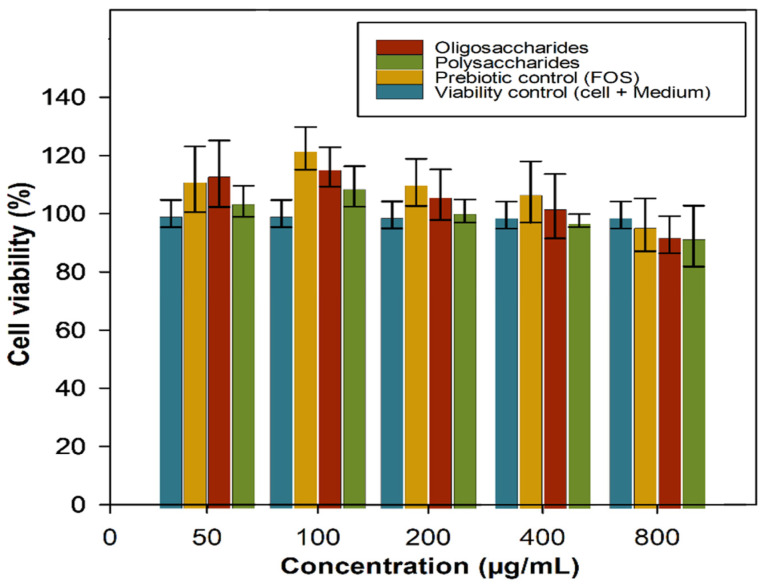
Cell viability of Caco-2 cells treated with different concentrations of polysaccharides, oligosaccharides, and FOS, compared with untreated control cells cultured in medium without treatment. Values are presented as mean ± standard deviation (*n* = 3), calculated from the average of three incubation times (24, 48, and 72 h). Statistical analysis was performed by one-way ANOVA, followed by Tukey’s post hoc test. No statistically significant differences were observed among the groups (*p* = 0.14).

Caco-2 cell viability remained above 90% across all evaluated concentrations (50 to 800 µg/mL) for both polysaccharide and oligosaccharide treatments. When all groups were analyzed together, the differences were not statistically significant (*p* = 0.14). Although polysaccharide treatments showed lower mean values at some concentrations, these differences were insufficient to indicate cytotoxicity. Across all experimental conditions, cells maintained high viability, as shown in Figure 3.

### 3.7. Growth of Probiotic Bacteria in MRS Broth Supplemented with Oligosaccharides and Polysaccharide Fibers

**Figure 4:**
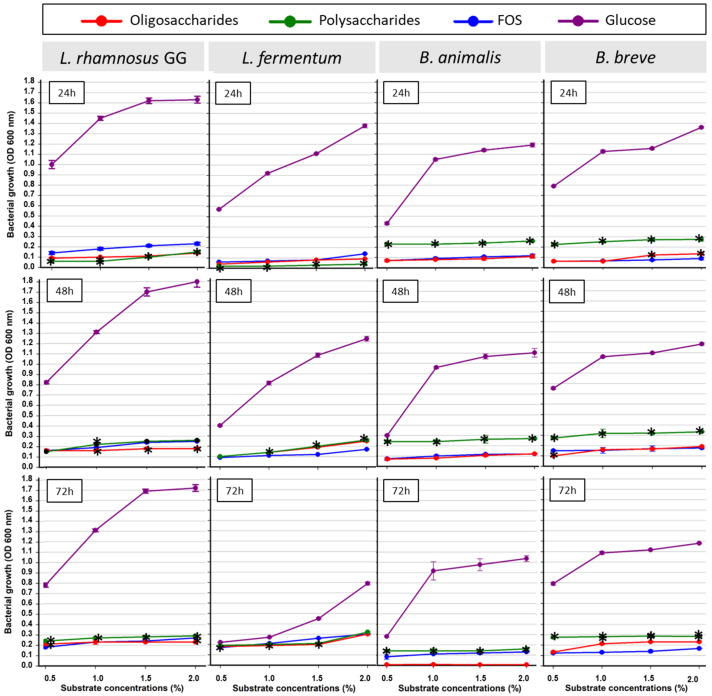
Growth (OD_600_) of *L. rhamnosus* GG, *L. fermentum*, *B. animalis*, and *B. breve* after 24, 48, and 72 h of anaerobic fermentation with oligosaccharides or polysaccharides extracted from *L. leucocephala* seeds at concentrations of 0.5, 1.0, 1.5, and 2.0% (*w*/*v*). FOS served as the reference prebiotic control and glucose as the rapidly fermentable carbon source. Data are presented as mean ± standard deviation (*n* = 3). Asterisks (*) indicate significant differences compared to FOS at the same time point and concentration (*p* < 0.05), as assessed by three-way repeated-measures ANOVA followed by Tukey’s post hoc test.

For all probiotic bacterial species evaluated, glucose supported rapid fermentation and produced the highest optical density values (OD_600_) across all concentrations and incubation times. In contrast, FOS, as well as oligosaccharides and polysaccharides derived from *L. leucocephala*, exhibited a slower fermentation profile, with gradual increases in bacterial growth over time (Figure 4).

For *L. rhamnosus* GG, oligosaccharides and polysaccharides supported a progressive increase in growth throughout the incubation period. At 48 h, polysaccharides reached OD_600_ values comparable to those of FOS at all tested concentrations, with a difference observed at 1.0% (*p* = 0.005). At 72 h, polysaccharides yielded higher OD_600_ values than FOS across all evaluated concentrations (0.5 to 2.0%, *p* ≤ 0.046).

For *L. fermentum*, at 48 h, oligosaccharides and polysaccharides resulted in higher OD_600_ values than FOS at concentrations of 1.0, 1.5 and 2.0% (*p* < 0.001), corresponding to the main growth phase for this species under the tested conditions.

For *B. animalis*, from 24 h onward, polysaccharides produced higher OD_600_ values than FOS at all concentrations tested (*p* < 0.001), whereas oligosaccharides showed OD_600_ values comparable to the prebiotic control. This pattern persisted at 48 h (*p* ≤ 0.002) and 72 h (*p* < 0.001).

For *B. breve*, polysaccharides produced higher OD_600_ values than FOS at all concentrations from 24 h onward (*p* < 0.001). Oligosaccharides showed values comparable to FOS at lower concentrations, but at 72 h, OD_600_ values were higher at 1.0, 1.5, and 2.0% (*p* < 0.001).

Collectively, the tested substrates exhibited distinct patterns of bacterial growth over time. Glucose supported higher optical density values during the initial incubation periods, followed by stabilization or a slight reduction at 72 h. In contrast, FOS, as well as oligosaccharides and polysaccharides from *L. leucocephala*, sustained bacterial growth throughout the incubation period. Polysaccharides yielded higher optical density values than FOS at multiple concentrations and time points, particularly for *Bifidobacterium* species.

### 3.8. Effect of Soluble Fibers on Pathogenic Microorganisms

**Figure 5:**
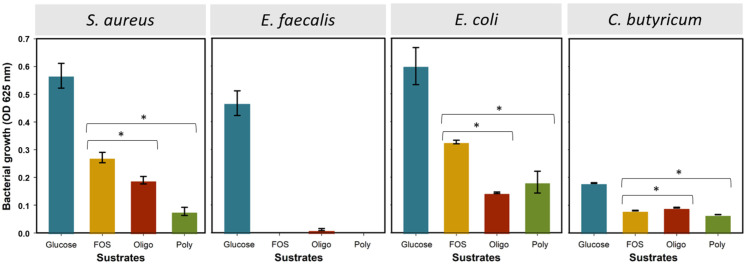
Growth (OD_625_) of *S. aureus*, *E. coli*, and *E. faecalis* after 24 h of aerobic incubation and *C. butyricum* after 48 h of anaerobic incubation in medium supplemented with glucose, FOS, oligosaccharides, or polysaccharides at 2.0% (*w*/*v*) concentration. Lower OD values indicate reduced bacterial growth. Data represent mean ± standard deviation (*n* = 3). Statistical comparisons were performed using one-way ANOVA followed by Dunnett’s post hoc test or the Kruskal–Wallis test with Dunn’s post hoc test, as appropriate. Asterisks (*) denote significant differences relative to FOS (*p* < 0.05).

Growth of pathogenic bacterial species varied by substrate when oligosaccharides and polysaccharides were compared with the prebiotic control, FOS (Figure 5). Pathogenic bacteria were evaluated at 2.0% (*w*/*v*), the highest fiber concentration tested, to assess bacterial growth under conditions that represent the greatest potential for growth stimulation. For *S. aureus*, optical density values were lower in cultures supplemented with oligosaccharides (*p* = 0.007) and polysaccharides (*p* < 0.001), with polysaccharides yielding lower values than oligosaccharides. In *E. coli*, oligosaccharides (*p* < 0.001) and polysaccharides (*p* = 0.002) also resulted in lower optical density values than FOS, with oligosaccharides showing the lowest values.

*E. faecalis* exhibited low optical density across all tested conditions, with comparable growth among substrates (*p* = 0.071). For *C. butyricum*, cultures supplemented with polysaccharides showed lower optical density than FOS (*p* < 0.001), whereas oligosaccharides yielded higher optical density (*p* < 0.001).

### 3.9. In Vitro Fermentation of Prebiotic Substrates by Probiotic Bacterial Species

**Figure 6:**
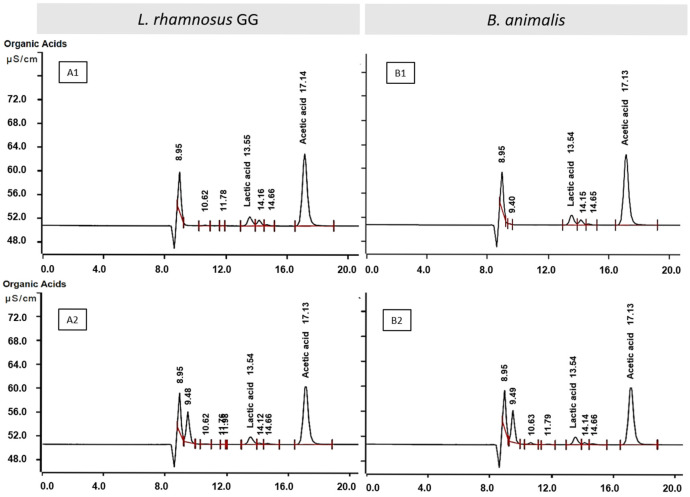
Representative ion chromatography profiles showing lactic acid and acetic acid peaks from fermentation supernatants of oligosaccharide and polysaccharide substrates by *L. rhamnosus* GG and *B. animalis*. (**A1**) *L. rhamnosus* GG fermented oligosaccharides. (**A2**) *L. rhamnosus* GG fermented polysaccharides. (**B1**) *B. animalis* fermented oligosaccharides. (**B2**) *B. animalis* fermented polysaccharides. Organic acids were quantified in fermentation supernatants after 24 h of anaerobic incubation with the substrates.

The in vitro fermentation of oligosaccharide and polysaccharide substrates by *L. rhamnosus* GG and *B. animalis* led to the detection of acetate and lactic acid in the fermentation supernatants (Table 5). Acetate was detected at higher concentrations than lactic acid under all tested conditions. For both substrates, *L. rhamnosus* GG exhibited higher concentrations of acetate and lactic acid than *B. animalis*. Representative chromatographic profiles for each fermentation condition are shown in Figure 6.

## 4. Discussion

### 4.1. Structural Behavior of Seed Fibers During Simulated Digestion

Simulated gastrointestinal digestion revealed differential resistance among fibers derived from the seed coat, cotyledons, and endosperm of *L. leucocephala*. While fibers from the seed coat and cotyledons exhibited surface erosion and increased porosity after digestion, endosperm polysaccharides largely retained a compact, continuous structure, indicating greater resistance to enzymatic degradation. Similar responses have been reported for non-starch polysaccharides [33].

The higher structural stability of endosperm fibers is consistent with the presence of high-molecular-weight galactomannans, whose branched, densely packed architecture limits enzyme accessibility and hydrolysis [34]. This resistance is a key feature of fibers that resist enzymatic digestion and are subsequently fermented by microbes.

Elemental analysis further supported digestion-induced compositional changes. The reduction in carbon content across all tissues reflects the removal of organic components, particularly soluble carbohydrates and proteins, whereas the enrichment of calcium in cotyledon and endosperm residues suggests exposure of wall-associated mineral forms following matrix hydrolysis [33,35]. In contrast, the depletion of magnesium and nitrogen in cotyledon residues likely reflects their association with soluble and protein-bound compounds that are transferred to the liquid phase during digestion. The appearance of potassium in the endosperm only after digestion may reflect the release of intracellular or vacuolar contents, increasing its detectability by surface EDS [35].

In terms of fiber yield, simulated digestion reduced the amount of endosperm polysaccharides, whereas oligosaccharides derived from the seed coat and cotyledons remained quantitatively stable. This behavior likely results from partial depolymerization of polysaccharides and migration of lower-molecular-weight fragments into the soluble phase [36,37].

Overall, the combined SEM, EDS, and yield data indicate that simulated digestion selectively removes digestible components while preserving a structurally relevant fibrous matrix, particularly in the endosperm. From a functional perspective, this resistance to human digestive enzymes is advantageous, as it promotes the arrival of *L. leucocephala* fibers in the colon, where they can be selectively fermented by the gut microbiota and contribute to short-chain fatty acid production and intestinal homeostasis [1,38]. Importantly, this digestion-resistant fibrous matrix was obtained from seeds subjected to moist-heat pretreatment (100 °C, 20 min) before simulated digestion. This condition is mild compared with the main thermal degradation range of legume galactomannans, which typically occurs between approximately 235 and 322 °C [39,40,41].

### 4.2. Rheological Behavior and Yield of Soluble Seed Fibers

The rheological behavior of *L. leucocephala* seed fibers after simulated digestion highlighted the endosperm as the most viscous component. This behavior is directly associated with the high content of viscous polysaccharides in the endosperm and is consistent with that of other galactomannan-rich legume seeds, such as guar and tamarind [16,42,43].

The pseudoplastic behavior of endosperm reflects the formation of a dense polymeric network, typical of galactomannan-rich matrices [42,44]. These rheological properties are relevant from a physiological perspective because highly viscous fibers are known to modulate digesta transit and substrate availability in the gastrointestinal tract.

The analysis of soluble fiber yield after simulated digestion further underscores the endosperm’s central role as the primary polysaccharide-rich component of *L. leucocephala* seeds, accounting for approximately 55% of the recovered soluble fiber. This distribution aligns with the anatomical organization of legume seeds, in which the endosperm serves as a reservoir of high-molecular-weight non-starch polysaccharides [42,45].

Taken together, high viscosity, pseudoplastic behavior, and elevated soluble fiber yield identify the endosperm as the primary functional fiber source in *L. leucocephala* seeds.

### 4.3. Carbohydrate Structure and Implications for Fermentability

Monosaccharide composition analysis indicated that the oligosaccharides derived from *L. leucocephala* seeds are predominantly composed of galactose, arabinose, and xylose, consistent with the presence of galactooligosaccharides (GOS) and arabinoxylo-oligosaccharides (AXOS) [38,46,47]. These carbohydrate structures are recognized as prebiotic substrates that resist digestion in the upper gastrointestinal tract and are selectively utilized by *Bifidobacterium* and *Lactobacillus* species, promoting beneficial microbial activity and short-chain fatty acid production.

In parallel, the polysaccharides contained mannose, galactose, xylose and arabinose, indicating the presence of galactomannans, xylans and arabinoxylans. Galactomannans, characterized by a β-(1→4)-mannan backbone substituted with α-(1→6)-linked galactose residues, are typical components of legume endosperms such as guar and carob [42,48]. These high-molecular-weight polysaccharides are not absorbed by the host and can be depolymerized by microbial enzymes, including β-mannanases and α-galactosidases, generating fermentable substrates that support bacterial growth throughout the colon [49].

Oligosaccharides and polysaccharides confer complementary fermentability dynamics, with GOS and AXOS supporting earlier microbial utilization, while galactomannans and arabinoxylans provide a more sustained source of fermentable carbohydrates through gradual depolymerization [49]. This complementarity can be interpreted through the enzymatic repertoire of probiotic bacteria: *Bifidobacterium* species encode β-galactosidases (GH families 2, 35, and 42) and solute-binding protein (SBP)-dependent ABC transporters that mediate the selective uptake and intracellular hydrolysis of GOS [50], whereas *Lactobacillus* species utilize phosphotransferase systems (PTS) and permeases for the internalization of shorter oligosaccharides [51]. For galactomannan utilization, bacterial β-mannanases (GH family 26) and α-galactosidases (GH family 27/36) catalyze the sequential depolymerization of the mannan backbone and its galactose side chains, generating mannooligosaccharides that serve as fermentable substrates [34,48]. The differential enzymatic capacity of probiotic and pathogenic species to access these structurally complex carbohydrates provides a molecular basis for the microbial selectivity observed in the present study. This structural diversity of galactomannans and soluble oligosaccharides demonstrates the adaptive resilience of *L. leucocephala* seeds, which maintain or increase soluble fiber content under environmental pressures in tropical environments [52].

### 4.4. Safety Assessment of Seed Fibers in Caco-2 Intestinal Epithelial Cells

Cell viability was maintained in Caco-2 intestinal epithelial cells exposed to oligosaccharides and polysaccharides derived from *L. leucocephala* at concentrations of 50 to 800 µg/mL and exposure periods of 24, 48, and 72 h. These findings indicate the absence of a cytotoxic response under the conditions evaluated, which is consistent with previous studies reporting that prebiotic fibers preserve epithelial viability [53].

Importantly, the thermal pretreatment (100 °C for 20 min) and solvent extraction protocol employed in this study effectively minimize antinutritional factors, including mimosine, a compound naturally present in *Leucaena* species and significantly reduced by these processing conditions [54,55,56]. Combined with the high cytocompatibility observed (>90% viability across all concentrations and time points), this processing approach confirms the safety profile of *L. leucocephala* seed fibers for food applications.

The viability of Caco-2 cells exposed to *L. leucocephala* polysaccharides and oligosaccharides across all time points underscores a favorable cytocompatibility profile comparable to that of FOS, supporting their suitability for functional food applications [29,57].

### 4.5. Selective Fermentation by Probiotic Bacteria and Production of Short-Chain Fatty Acids

Fermentation assays showed that oligosaccharides and polysaccharides derived from *L. leucocephala* supported the growth of all tested probiotic bacterial species, with optical density values comparable to or exceeding those of the FOS control at specific concentrations. This response is consistent with the presence of GOS, AXOS, and galactomannan-related structures, which are efficiently metabolized by *Bifidobacterium* and *Lactobacillus* species through the action of carbohydrate-active enzymes such as β-galactosidases, β-mannanases, and endoxylanases [31,51].

Polysaccharides showed a slower, more sustained fermentation profile over 24 to 72 h, reflecting the gradual enzymatic breakdown of more complex structures. In contrast, the lower OD values observed for *L. fermentum* at 72 h may reflect earlier substrate depletion and the accumulation of fermentation metabolites, leading to a transition to the stationary phase. This behavior reflects species-specific differences in metabolic activity and tolerance to prolonged fermentation conditions, as reported for lactic acid bacteria [58,59]. In addition, *L. fermentum*, a heterofermentative species, exhibits faster initial substrate utilization and earlier entry into the stationary phase than other *Lactobacillus* species [60].

Analysis of fermentation metabolites revealed acetate as the predominant short-chain fatty acid produced, along with lactate accumulation during fermentation by *L. rhamnosus* GG and *B. animalis* subsp. *lactis*. This metabolic pattern is characteristic of lactic acid bacteria and bifidobacteria, whose carbohydrate catabolism primarily yields acetate and lactate via the fructose-6-phosphate phosphoketolase pathway [61,62]. The absence of butyrate and propionate in these monoculture systems is expected, as these metabolites are produced by secondary fermenters that rely on cross-feeding interactions in complex microbial communities [63,64].

Acetate is the most abundant SCFA in the colon and exerts biological effects through at least two established molecular pathways: (i) activation of G-protein-coupled receptors GPR43 (FFAR2) and GPR41 (FFAR3) expressed on intestinal epithelial cells, enteroendocrine L-cells, and immune cells, triggering intracellular signaling cascades that regulate inflammatory responses and promote glucagon-like peptide-1 (GLP-1) secretion [6,65], and (ii) inhibition of histone deacetylases (HDACs), modulating gene expression related to epithelial barrier integrity and antimicrobial peptide production [8,65]. Although not classified as an SCFA, lactate is a key metabolic intermediate in microbial cross-feeding networks. It serves as a substrate for butyrate-producing species, such as *Faecalibacterium prausnitzii* and *Roseburia intestinalis*, via the butyryl-CoA:acetate CoA-transferase pathway and for propionate-producing species via the acrylate pathway [63,64]. Thus, the acetate- and lactate-rich fermentation profile generated by *L. leucocephala* fibers in monoculture provides a metabolic foundation that, within a complex colonic ecosystem, could support the downstream production of butyrate and propionate through established cross-feeding routes.

Taken together, the fermentation profile of *L. leucocephala* fibers is consistent with the selective utilization by lactic acid bacteria and bifidobacteria, with acetate and lactate as the primary outputs. Although these metabolites are not the end products most relevant to colonic physiology (butyrate and propionate), they constitute the substrates from which secondary fermenters generate acids in complex communities. The acetate- and lactate-rich profile observed here in monoculture is therefore the expected upstream signature of a fiber with prebiotic potential under co-culture or in vivo conditions.

### 4.6. Lack of Growth Stimulation in Pathogenic Bacteria

Pathogenic bacterial species showed limited growth when cultured with oligosaccharides and polysaccharides derived from *L. leucocephala*. This pattern indicates that these microorganisms were unable to efficiently utilize *L. leucocephala* fibers as carbon sources, reflecting a limited enzymatic capacity to degrade structurally complex carbohydrates [49,51].

The limited growth of *S. aureus*, *E. coli*, and *E. faecalis* in culture medium supplemented with *L. leucocephala* fibers reflects the restricted repertoire of carbohydrate-active enzymes (CAZymes) in these species. Genomic analyses have shown that pathogenic bacteria typically lack the glycoside hydrolase families required for the depolymerization of GOS (GH2, GH42), galactomannans (GH26, GH27/36), and arabinoxylans (GH10, GH11, GH43), which are abundantly encoded in the genomes of *Bifidobacterium* and *Lactobacillus* species [49,50,51]. Furthermore, pathogenic species lack specialized oligosaccharide transport systems, including SBP-dependent ABC transporters and MFS permeases, which enable probiotic bacteria to internalize and metabolize complex prebiotic structures [51]. This enzymatic and transport asymmetry between probiotic and pathogenic species constitutes the molecular basis for the microbial selectivity observed in the present study, consistent with the ISAPP criterion of selective utilization [1,66].

For *C. butyricum*, growth in culture medium supplemented with *L. leucocephala* fibers was lower than that observed with fructooligosaccharides, indicating a greater degree of metabolic incompatibility with these substrates. This behavior aligns with reports indicating that *C. butyricum* has a limited capacity to metabolize plant non-starch polysaccharides and prebiotic oligosaccharides [49,67].

Collectively, the present findings distinguish this study from previous reports on in vitro prebiotic evaluation in three key aspects. First, *L. leucocephala* seed-derived fibers represent a previously uncharacterized source for human prebiotic applications, as prior research on this species has focused on ruminant nutrition. Second, unlike studies that evaluate digestibility, fermentation, or safety in isolation, this study combines INFOGEST 2.0 gastrointestinal simulation, rheological profiling, SEM–EDS structural analysis, GC-MS monosaccharide composition, dual fermentation assessment with both probiotic and pathogenic species, metabolite quantification, and Caco-2 cytotoxicity within a unified experimental framework. Third, the anatomical specific approach, which separates the seed coat, cotyledon, and endosperm, identified the endosperm as the primary source of functional fiber. This level of resolution is not commonly applied in the prebiotic screening of novel plant matrices. The development of a patented synthetic medium for pathogenic bacteria testing (BR 10 2025 025261-9) further represents a methodological contribution that enables the controlled evaluation of prebiotic selectivity without interference from complex nutrients.

### 4.7. Limitations and Future Perspectives

This study exclusively employed in vitro methodologies, including INFOGEST 2.0 gastrointestinal simulation, monoculture fermentation, and Caco-2 cytotoxicity assays. Although these approaches do not replicate the full complexity of in vivo conditions, including peristalsis, mucosal absorption, hormonal regulation, immune signaling, and microbial community interactions, they constitute the recognized initial step in the systematic evaluation of novel prebiotic candidates. According to the ISAPP expert framework for classifying compounds as prebiotics, establishing prebiotic status follows a stepwise process that begins with demonstrating digestive resistance and selective microbial utilization in vitro, before proceeding to in vivo validation and clinical trials [1,66]. This approach has been widely adopted to evaluate emerging prebiotic substrates from diverse botanical sources [16,17,18].

Within this framework, several limitations must be acknowledged. The INFOGEST static digestion model reproduces key gastrointestinal physicochemical conditions but does not account for dynamic processes such as peristalsis, nutrient absorption, or microbial feedback, which may lead to the accumulation of soluble digestion products and influence fiber yield estimates and compositional profiles [68]. Therefore, future studies should incorporate dynamic or semi-continuous digestion models to better simulate intestinal transit and absorption.

Fermentation assays conducted under monoculture conditions assess substrate-specific utilization by individual bacterial species but do not capture the microbial interactions, including cross-feeding and syntrophic metabolism, that shape short-chain fatty acid profiles in vivo [64,69]. Notably, butyrate and propionate production in the gut depends primarily on secondary fermenters, such as *F. prausnitzii* and *Roseburia* spp., which were not included in the present design [63,64]. To address this limitation, future studies should employ fecal inoculum-based fermentation systems and continuous colonic models (e.g., SHIME and TIM-2) to evaluate microbial succession, metabolite distribution, and community-level responses to *L. leucocephala* fibers.

In addition, interindividual variability in diet, gut microbiota composition, and host genetics may influence prebiotic responses, thereby affecting both fermentation outcomes and physiological effects [2,6]. The translation of the present in vitro findings will require validation in animal models to assess intestinal transit, full SCFA profiles (including butyrate and propionate), microbiota modulation by 16S rRNA gene sequencing, and histological evaluation of intestinal barrier integrity. Subsequently, randomized controlled human intervention trials will be essential to establish health-relevant outcomes and confirm the prebiotic status of *L. leucocephala* fibers according to the ISAPP criteria [1,66].

From a mechanistic perspective, future studies should employ transcriptomic or metatranscriptomic approaches to identify the specific carbohydrate-active enzymes (CAZymes) and transport systems that are upregulated during the fermentation of *L. leucocephala* fibers by probiotic bacteria. In addition, GPR43/GPR41 receptor activation assays using Caco-2 or HT-29 reporter cell lines exposed to fermentation supernatants would enable the functional characterization of the signaling pathways activated by acetate and lactate produced during fermentation. These approaches would provide the mechanistic resolution necessary to complement the phenotypic evidence presented in this study.

The standardized INFOGEST digestion and targeted fermentation assays used here align with current recommendations for the preclinical evaluation of candidate prebiotics and can reduce the scope of subsequent animal experiments by triaging promising substrates before in vivo studies. Within this framework, the *L. leucocephala* dataset functions as a decision-support tool: it identifies the endosperm as the seed compartment that warrants further investigation, distinguishes it from the coat and cotyledon, and quantifies the level of selectivity that should be expected when these fibers are tested against complex microbial communities. Mechanistic dissection, CAZyme expression profiling, transporter activity, and SCFA receptor signaling are logical next steps but lie beyond the scope of the present in vitro phenotypic screen.

## 5. Conclusions

This study provides the first compartment-resolved assessment of the prebiotic potential of *L. leucocephala* seed fiber following INFOGEST 2.0 digestion. Anatomical dissection localized the digestion-resistant, viscous soluble fiber fraction to the endosperm, which retained its structural integrity after digestion and exhibited pronounced pseudoplastic behavior. GC-MS profiling revealed the presence of galacto- and arabinoxylo-oligosaccharides in the oligosaccharide pool and galactomannans, xylans, and arabinoxylans in the polysaccharide pool.

Both fractions were selectively fermented by *L. rhamnosus* GG, *L. fermentum*, *B. animalis* subsp. *lactis*, and *B. breve*, with growth equal to or exceeding that supported by fructo-oligosaccharides at multiple time points and at multiple concentrations. The same substrates did not support the growth of *S. aureus*, *E. coli*, *E. faecalis*, or *C. butyricum*, a selectivity that was tested in a synthetic medium developed for this purpose (Brazilian patent application BR 10 2025 025261-9). Acetate and lactate were the dominant fermentation outputs, consistent with substrate feeding downstream of butyrate- and propionate-producing communities. Caco-2 viability remained above 90% for all concentrations and exposure times.

Together, these results identify *L. leucocephala* endosperm as a candidate source of prebiotic fiber and provide the in vitro evidence required for the first stage of the ISAPP framework. Confirmation of prebiotic status will require validation in animal models to assess transit, full SCFA profiles, microbiota modulation, and intestinal barrier integrity, followed by randomized human trials. The pipeline used in this study, including the patented synthetic medium for selectivity testing, is applicable to other underutilized tropical legumes.

## 6. Patents

A synthetic culture medium for the growth of pathogenic bacteria in prebiotic tests was developed and filed as a patent application during this research. This culture medium, described in detail in Section 2.8 (In vitro fermentation assay with pathogenic microorganisms), enables the controlled evaluation of prebiotic fibers’ effects on intestinal pathogens (*Escherichia coli* ATCC 25922, *Staphylococcus aureus* ATCC 25923, *Enterococcus faecalis* ATCC 29212, and *Clostridium butyricum* ATCC 860) without interference from complex nutrients found in conventional broths like BHI.

Patent details: Title: Caldo sintético para bactérias patogênicas em testes com prebióticos (Synthetic medium for pathogenic bacteria in prebiotic assays); application number: BR 10 2025 025261-9; filing date: 18 November 2025; petition number: 870250105384; depositant: Universidade Federal do Maranhão (UFMA); and inventors: Aleania Polassa Almeida Pereira, Allysson Kayron de Carvalho Silva, João Lucas do Carmo Lima, Viviane da Silva Sousa Almeida, Valério Monteiro-Neto.

This formulation was crucial to validate the results of selective fermentation (Section 3.8 and Figure 5), confirming that *L. leucocephala* fibers do not promote pathogen growth, unlike glucose or FOS controls (*p* < 0.05).

## Figures and Tables

**Table 1:** Rheological parameters of digested fluids fitted to the Ostwald–de Waele (Power Law) model.

Samples	Coefficients		*R* ^2 c^	MRE (%) ^d^
	*K* ^a^	*N* ^b^		
Seed Coat	28.863	0.335	0.848	14.363
Cotyledon	120.000	0.001	0.962	8.695
Endosperm	574.662	0.715	0.974	4.416
FOS (Control)	0.976	1.006	0.510	0.646

^a^: Consistency index (*k*), representing the resistance of the fluid to flow. ^b^: Flow behavior index (*n*), where *n* < 1 indicates pseudoplastic, *n* = 1 Newtonian, and *n* > 1 dilatant behavior. ^c^: Coefficient of determination (*R*^2^), expressing the fit quality of the Power Law model. ^d^: Mean relative error (MRE), with values below 10% indicating good model fit and values between 10 and 15% considered acceptable.

**Table 2:** Yield of soluble fiber obtained from the endosperm, seed coat, and cotyledon of *L. leucocephala* seeds before and after simulated digestion.

Anatomical Part	Mass (g)	Fiber Type	Fiber Mass (g) Before → After	Fiber Yield (%) Before → After ^a^	*p*-Value
Endosperm	1.00	Polysaccharides	0.62 → 0.56	62.0 → 56.0	0.025
Seed coat	1.00	Oligosaccharides	0.10 → 0.09	10.8 → 9.0	0.311
Cotyledon	1.00	Oligosaccharides	0.001 → 0.001	0.2 → 0.2	0.326

^a^: Fiber yields were calculated as the extracted fiber mass relative to the initial 1 g of dry sample before and after simulated digestion. Values represent the mean of three replicates (*n* = 3). A Student’s *t*-test was used to compare values before and after digestion. Differences were considered statistically significant at *p* < 0.05.

**Table 3:** Elemental composition expressed as weight percentage (wt%) of each element relative to the total detected mass before and after simulated digestion.

Element	Group	Undigested ^a^	Digested ^a^	*p*-Value
Carbon	Seed coat	42.56 ± 0.70	12.86 ± 2.93	<0.001
	Cotyledon	29.52 ± 1.83	3.21 ± 1.24	<0.001
	Endosperm	37.62 ± 2.66	14.18 ± 2.23	<0.001
Calcium	Seed coat	10.68 ± 1.05	21.49 ± 0.98	<0.001
	Cotyledon	7.45 ± 1.87	ND	0.002
	Endosperm	2.98 ± 1.69	15.73 ± 1.99	0.001
Potassium	Seed coat	2.51 ± 0.34	3.77 ± 0.43	0.016
	Cotyledon	ND	ND	-
	Endosperm	ND	4.25 ± 0.59	<0.001
Oxygen	Seed coat	3.07 ± 0.70	1.29 ± 0.08	0.012
	Cotyledon	1.44 ± 0.28	ND	<0.001
	Endosperm	0.63 ± 0.35	1.27 ± 0.41	0.109
Magnesium	Seed coat	0.92 ± 0.19	1.35 ± 0.02	0.016
	Cotyledon	0.76 ± 0.56	ND	0.089
	Endosperm	ND	ND	-
Nitrogen	Seed coat	ND	ND	-
	Cotyledon	5.14 ± 1.12	ND	0.001
	Endosperm	2.49 ± 0.93	2.08 ± 0.57	0.543

^a^: Values are expressed as mean ± standard deviation (wt%). Statistical differences between undigested and digested samples were assessed using Student’s *t*-test within each anatomical part. ND indicates elements not detected by SEM-EDS in both conditions. When an element was detected in only one condition, a nominal value of 0.01 wt% was used exclusively for statistical analysis to enable comparison between undigested and digested samples. A dash (-) indicates cases in which statistical analysis could not be performed because the element was not detected in either condition. Differences were considered statistically significant at *p* < 0.05.

**Table 4:** Monosaccharide profile and structural inference of oligosaccharides and polysaccharides.

Fibers	Identified Monosaccharides	RT (min)	Inferred Structure
Oligosaccharide	Galactose	28.5	GOS
	Arabinose	17.4	AXOS
	Xylose	20.5	
Polysaccharide	Mannose	26.5	Galactomannans
	Galactose	28.5	
	Xylose	20.5	Xylans
	Xylose	20.5	Arabinoxylans
	Arabinose	17.4	

RT: retention time in GC-MS analysis; GOS: galactooligosaccharides; AXOS: arabinoxylooligosaccharides. Values based on GC-MS after acid hydrolysis and derivatization.

**Table 5:** Concentrations of lactic acid and acetate (ppm) in fermentation supernatants obtained after in vitro fermentation of oligosaccharide and polysaccharide substrates by *L. rhamnosus* GG and *B. animalis*.

Bacterium	Substrate	Lactic Acid (ppm)	Acetate (ppm)
*L. rhamnosus* GG	Oligosaccharide	1035 ± 4.4	4789 ± 19.6
*L. rhamnosus* GG	Polysaccharide	1087 ± 28.9	4591 ± 108.6
*B. animalis*	Oligosaccharide	909 ± 14.1	3990 ± 47.6
*B. animalis*	Polysaccharide	818 ± 7.5	3846 ± 17.3

Ppm: parts per million. Values are expressed as mean ± standard deviation (*n* = 3).

## Data Availability

The raw data supporting the conclusions of this article will be made available by the authors on request.

## Abbreviations

The following abbreviations are used in this manuscript:

AXOS	Arabinoxylo-oligosaccharides
BCF	Benzyl chloroformate
BHI	Brain heart infusion
cP	Centipoise
CFU	Colony-forming units
DMSO	Dimethyl sulfoxide
EDS	Energy-dispersive X-ray spectroscopy
FBS	Fetal bovine serum
FOS	Fructooligosaccharides
GC-MS	Gas chromatography-mass spectrometry
GOS	Galactooligosaccharides
INFOGEST	International Network on Food Digestion
MH	Mueller-Hinton
MRS	Man, Rogosa, and Sharpe
MTT	3-(4,5-dimethylthiazol-2-yl)-2,5-diphenyltetrazolium bromide
OD	Optical density
ppm	Parts per million
rpm	Revolutions per minute
SCFAs	Short-chain fatty acids
SEM	Scanning electron microscopy
SGF	Simulated gastric fluid
SIF	Simulated intestinal fluid
SSF	Simulated salivary fluid
TFA	Trifluoroacetic acid
TMSI	N-trimethylsilylimidazole
*w/v*	Weight/volume
